# The 100 Top-Cited Studies on Neuropsychology: A Bibliometric Analysis

**DOI:** 10.3389/fpsyg.2020.550716

**Published:** 2020-11-30

**Authors:** Yang Zhang, Ying Xiong, Yujia Cai, Linli Zheng, Yonggang Zhang

**Affiliations:** ^1^Department of Periodical Press and National Clinical Research Center for Geriatrics, West China Hospital, Sichuan University, Chengdu, China; ^2^Chinese Evidence-Based Medicine Center, West China Hospital, Sichuan University, Chengdu, China; ^3^Mental Health Center, West China Hospital, Sichuan University, Chengdu, China

**Keywords:** neuropsychology, top-cited, bibliometric analysis, citation, citation analysis

## Abstract

**Objective:**

The present study aimed to identify and analyze the bibliometric characteristics of the 100 top-cited studies on neuropsychology.

**Methods:**

We searched the Web of Science Core Collection database to collect studies on neuropsychology from inception to 31st December 2019. Two authors independently screened the literature and extracted the data. Statistical analyses were performed using R software.

**Results:**

The 100 top-cited articles were cited a total of 166,123 times, ranging from 736 to 24,252 times per article. All of the studies were published from 1967 to 2014 in 47 journals. *Neuropsychologia* had the highest number of articles (*n* = 17), followed by *Neurology* (*n* = 8). The top three most productive countries were the USA (*n* = 60), England (*n* = 13), and Canada (*n* = 8). Eight authors contributed the same number of studies as the first author (*n* = 2) or corresponding author (*n* = 2). The most productive institute was the University of California (*n* = 9), followed by the University of Pennsylvania (*n* = 4). Of the 100 top-cited publications, 64 were original articles, and 36 were reviews. The top three Web of Science categories were clinical neurology (*n* = 28), behavioral sciences (*n* = 19), and psychiatry (*n* = 11).

**Conclusion:**

This study provides insight into the impact of neuropsychology research and may help doctors, researchers, and stakeholders to achieve a more comprehensive understanding of trends and most influential contributions to the field, thus promoting ideas for future investigation.

## Introduction

Neuropsychology was recognized as a discipline distinct from applied areas of psychology or neurology in the 1960s (Klove, 1963). Although ideas about the connection between behavior and brain function can be traced back to Pythagoras, systematic studies of brain–behavior relationships only began in the 19th century. Neuropsychology research encompasses both healthy and disordered states, such as consciousness (Berlucchi and Marzi, 2019), aging (Lu and Lee, 2017; Cohen et al., 2019), memory (Ruchinskas and Cullum, 2018), brain injury (Prince and Bruhns, 2017), and Alzheimer’s disease (Pena-Casanova et al., 2012). With advances in neuroimaging, the human genome project, theories of psychological testing, and information technology, neuropsychological research contents and methods have expanded (Bilder, 2011). The number of neuropsychology studies published each year is increasing, thus necessitating a bibliometric analysis of existing research.

In bibliometric analysis, quantitative analysis techniques are used to examine published literature and identify the characteristics of influential studies (King, 2004). Bibliometric analysis enables external evaluation of research quality, impact and prestige factors, and examination of development within a field of study. By exploring published findings using robust criteria for standardization of academic quality, bibliometric analysis can be valuable in identifying trends in research and fostering ideas for future study (Hirsch, 2005; Bar-Ilan, 2008). One of the most important bibliometric analysis methods is citation analysis, which provides comprehensive information about cited articles (Shadgan et al., 2010; Ho, 2012). The frequency of citation indicates the relative significance of an article in a particular discipline, and identifying the top-cited studies in a field can help to understand research achievements and guide investigators toward new directions. Moreover, it can provide insights about the main interests in certain fields, with an emphasis on the journals and authors that have the greatest impact worldwide.

Citation analysis has been applied to various research fields, such as diabetes (Yang et al., 2020), traumatic brain injury (Sharma and Lawrence, 2014), medical education (Azer, 2016), vaccines (Zhang et al., 2019b), radiology (Mauri and Belli, 2015), cancer (Powell et al., 2016), etc. Bibliometric analysis highlights the key topics and publications that have shaped the understanding and management of certain fields. However, no bibliometric analyses have examined the field of neuropsychology. Analysis of these data provides insight into how our understanding of neuropsychology developed. Additionally, this work provides evidence-based reference for the most cited papers in neuropsychology. Therefore, in the present study, we aimed to identify and analyze the bibliometric characteristics of the 100 top-cited publications in neuropsychology.

## Materials and Methods

### Search Strategy

We searched the Web of Science Core Collection database to collect studies on neuropsychology from inception to 31st December 2019. We used the following search strategies TS = (neuropsycholog^∗^) OR SO = (neuropsycholog^∗^), where TS represents “topic search” and includes the title, abstracts, author keywords, and keywords, and “SO” stands for the journal name. The retrieved articles were listed in descending order according to the total number of citations. If two articles had the same number of citations, the article that was published more recently was ranked higher. Articles that were published in English, in indexed journals, and that focused on psychological issues from the perspective of neuroscience were included in the analysis. Articles that only mentioned brain activity or that focused on behavior from a purely psychological perspective were excluded. Editorials, letters, proceedings, meeting reports, and books were also excluded. Two authors independently screened the title, abstract, and full text of every publication in the list until they identified the 100 most frequently cited studies. Disagreements were resolved *via* discussion. If necessary, discrepancies were resolved by the third author. The resulting 100 top-cited articles in neuropsychology were then subjected to further analysis.

### Data Extraction

Two authors extracted the data independently. Information including the title, total number of citations, publication year, average citations per annum since the publication year, first author, corresponding author, country and institution of the corresponding author, article type, Web of Science category, journal name, and journal impact factor according to a journal citation report from 2018 was identified and recorded. If an article had more than one corresponding author, affiliation, or category, the first was record.

### Statistical Analysis

We used the R software from the “The Comprehensive R Archive Network” to perform correlation analysis. Correlation analysis was used to evaluate relationships between the different terms. *P* < 0.05 (two-tailed) was considered statistically significant. VOSviewer 1.6.6 (Leiden University)^1^ was used to analyze the co-authorship and co-citation of the top 100 studies.

## Results

### Basic Characteristics of the 100 Top-Cited Studies

Supplementary Table S1 lists the 100 top-cited publications in neuropsychology according to the total number of citations, ranked in descending order. The 100 top-cited articles were cited a total of 166,123 times with a median citation number of 1,661, ranging from 736 to 24,252. The most frequently cited article, which received 24,252 citations, was published in *Neuropsychologia* in 1971 (Oldfield, 1971). The study proposed a simple and brief method of assessing handedness on a quantitative scale for use in neuropsychological, clinical, and experimental research. The incidence of handedness between the sexes is also discussed in the study (Oldfield, 1971). The average number of citations per year for the 100 top-cited articles ranged from 16 to 666, with a median of 92 citations. The annual average number of citations of the 100 articles was correlated with the total number of citations (*r* = 0.676, *P* < 0.01).

### Journals, Publication Year, and Country

The 100 top-cited studies were published in 47 journals. The journals that published at least two of the studies are listed in Table 1. *Neuropsychologia* had the highest number of publications (*n* = 17), followed by *Neurology* (*n* = 8) and *Psychological Review* (*n* = 5). *American Journal of Psychiatry*, *Archives of Neurology* (*now JAMA Neurology*), *New England Journal of Medicine*, and *Archives of General Psychiatry* (*now JAMA Psychiatry*) published the same number of articles (*n* = 4). The other 40 journals published less than 4 articles from the list. The impact factor of the journals that published at least two of the 100 top-cited studies ranged from 1.344 to 70.670. We did not find a relationship between the number of studies published and the impact factor of the journal (*P* > 0.05). The average number of citations for each article was not correlated with the journal’s impact factor (*P* > 0.05). The representative journals of two biggest clusters of cited sources were *Neuropsychologia* and *Neurology*. The co-citation map of cited sources is shown in Supplementary Figure S1.

**TABLE 1:** Journals that published at least two of the 100 top-cited studies.

**Journal**	**Total citation**	**Number of studies**	**Average citation**	Impact factor (2018)*
Neuropsychologia	39,003	17	2,294	2.872
Neurology	15,685	8	1,961	8.689
Psychological Review	10,617	5	2,123	6.266
American Journal of Psychiatry	7,541	4	1,885	13.655
Archives of Neurology (now JAMA Neurology)	6,564	4	1,641	12.321
New England Journal of Medicine	4,707	4	1,177	70.670
Archives of General Psychiatry (now JAMA Psychiatry)	3,419	4	855	15.916
Brain	3,471	3	1,157	11.814
Alzheimer’s and Dementia	8,357	2	4,179	14.423
Psychological Bulletin	5,001	2	2,501	16.405
Cognition	4,374	2	2,187	3.537
Trends in Cognitive Sciences	4,217	2	2,109	16.173
Trends in Neurosciences	4,263	2	2,132	12.314
Stroke	2,482	2	1,241	6.058
Lancet	2,212	2	1,106	59.102
Neuron	2,103	2	1,052	14.403
JAMA—Journal of the American Medical Association	1,835	2	918	51.273
Lancet Neurology	1,823	2	912	28.755
Neuropsychology Review	1,672	2	836	5.739
Developmental Neuropsychology	1,599	2	800	1.344

*Impact factor was from the journal citation report of 2018.

All of the studies were published between 1967 and 2014 (Table 2). The year in which the highest number of top-cited articles was published was 1999 (*n* = 8), followed by 2004 (*n* = 7). Of all of the years examined, 1971 had the most citations (*n* = 24,252). There were 12 countries that contributed to the 100 top-cited publications (Table 3). The three most productive countries were the USA (*n* = 60), England (*n* = 13), and Canada (*n* = 8). Australia contributed five studies, whereas the other countries contributed no more than three publications each. The main partners of the USA were England, Canada, and Netherlands. The co-authorship map of countries is shown in Supplementary Figure S2.

**TABLE 2:** Publication year of the 100 top-cited studies.

**Year**	**Number of studies**	**Total citation**	**Average citation**
1967	1	910	910
1968	2	1,979	990
1971	1	24,252	24,252
1976	1	933	933
1982	1	936	936
1987	1	1,201	1,201
1989	1	2,635	2,635
1990	2	1,646	823
1991	5	8,262	1,652
1992	4	7,775	1,944
1993	3	10,737	3,579
1994	2	4,149	2,075
1995	2	1,912	956
1996	3	2,587	862
1997	4	7,117	1,779
1998	6	8,561	1,427
1999	8	8,353	1,044
2000	5	8,269	1,654
2001	6	7,795	1,299
2002	6	8,704	1,451
2003	6	9,136	1,523
2004	7	7,168	1,024
2006	4	3,349	837
2007	6	7,530	1,255
2008	2	1,716	858
2009	1	1,242	1,242
2010	1	1,212	1,212
2011	5	12,642	2,528
2012	1	947	947
2013	2	1,698	849
2014	1	770	770

**TABLE 3:** Countries of the 100 top-cited studies.

**Country**	**Number of studies**	**Total citation**	**Average citation**
USA	60	92,436	1,541
England	13	19,050	1,465
Canada	8	12,791	1,599
Australia	5	4,467	893
France	3	3,284	1,095
Scotland	2	25,039	12,520
Denmark	2	2,351	1,176
Netherlands	2	2,306	1,153
Italy	2	1,986	993
Norway	1	849	849
Israel	1	806	806
Spain	1	758	758

### Authors and Institutes

The authors who published at least two top-cited papers as a first author or corresponding author are listed in Table 4. There were eight authors who contributed the same number of studies as the first author (*n* = 2) or corresponding author (*n* = 2). The co-citation map of authors is shown in Supplementary Figure S3.

**TABLE 4:** Authors who published at least two papers as the first author or corresponding author.

**Author**	**Name**	**Number of studies**
First author	Baddeley, A	2
	Cummings, JL	2
	Heaton, RK	2
	McKhann, GM	2
	Petersen, RC	2
	Rorden, C	2
	Tombaugh, TN	2
	Saykin, AJ	2
Corresponding author*	Baddeley, A	2
	Cummings, JL	2
	Heaton, RK	2
	McKhann, GM	2
	Petersen, RC	2
	Rorden, C	2
	Tombaugh, TN	2

*If a study had more than one corresponding author, the first was used for data analysis.

A total of 16 institutes from four countries contributed more than two publications each (Table 5). The most productive institute was the University of California (*n* = 9), followed by the University of Pennsylvania (*n* = 4). Four institutes contributed three publications each, and 10 institutes contributed two studies each. Of the 16 institutions, 10 institutions were from the USA, and 14 were from the universities.

**TABLE 5:** Institutes that published at least two of the 100 top-cited studies.

Institute*	**Country**	**Number of studies**	**Total citation**
University of California	USA	9	11,549
University of Pennsylvania	USA	4	4,556
Johns Hopkins University	USA	3	7,626
University of Bristol	England	3	6,212
University of Lowa	USA	3	5,384
Harvard University	USA	3	5,196
Mayo Clinic	USA	2	4,023
Carleton University	Canada	2	3,996
Washington University	USA	2	3,619
University College London	England	2	2,202
University of North Carolina	USA	2	2,121
Columbia University	USA	2	2,175
University of Cambridge	England	2	1,906
McGill University	Canada	2	1,729
Vanderbilt University	USA	2	1,666
Austin Health	Australia	2	1,608

*The institute of the first corresponding author was used for data analysis.

### Study Type and Web of Science Categories

Of the 100 top-cited publications, 64 were original articles, and 36 were reviews (Table 6). The average number of citations for original articles (*n* = 1,717) was higher than that for reviews (*n* = 1,562). The 100 top-cited studies were divided into 15 Web of Science categories (Table 6). The top three Web of Science categories were clinical neurology (*n* = 28), behavioral sciences (*n* = 19), and psychiatry (*n* = 11).

**TABLE 6:** Study type of the top-cited studies.

**Variable**	**Number of study**	**Total citation**	**Average citation**
**Type of study**			
Article	64	109,892	1,717
Review	36	56,231	1,562
Web of Science categories*			
Clinical Neurology	28	49,590	1,771
Behavioral Sciences	19	43,220	2,275
Psychiatry	11	13,293	1,208
Neurosciences	10	12,386	1,239
Psychology	9	18,975	2,108
Medicine, General and Internal	8	8,754	1,094
Psychology, Clinical	5	4,623	925
Psychology, Experimental	2	4,374	2,187
Psychology, Developmental	2	1,599	800
Geriatrics and Gerontology	1	2,726	2,726
Linguistics	1	2,157	2,157
Gastroenterology and Hepatology	1	1,268	1,268
Rheumatology	1	1,178	1,178
Psychology, Mathematical	1	1,177	1,177
Biology	1	803	803

*We used the Web of Science categories. If an article had more than one category, the first was used for data analysis.

## Discussion

Although bibliometric analysis has been used to examine various research fields (Shadgan et al., 2010; Ho, 2012; Zhang et al., 2019b), few citation analyses have focused on neurology (Sharma and Lawrence, 2014; Lucena et al., 2019) or psychiatry (Trushchelev, 2012; Mazhari, 2013). To the best of our knowledge, no citation analyses have examined publications on neuropsychology. Therefore, this study represents the first comprehensive analysis of published literature in the field of neuropsychology.

In the present study, we found that the total number of citations of the 100 top-cited articles was 166,123, ranging from 736 to 24,252. The average citations per year ranged from 16 to 666, and the annual average number of citations of the 100 articles was correlated with the total number of citations. All of the studies were published from 1967 to 2014, and the year with the highest number of top-cited articles published was 1999. The list of top-cited articles was dominated by older articles. This is likely because articles published after 2014 were not included, and older articles are more likely to have a higher number of citations. Similar findings were reported in previous studies (Du et al., 2019; Zhang et al., 2019b); they also found that the top-cited articles in the fields of pain and depression and vaccine were not in recent years. From the perspective of the total number of citations, it is difficult to obtain high total citations for newly published articles due to the short time.

*Neuropsychologia* had the highest number of publications, followed by *Neurology*. They were representative journals of two biggest clusters of cited sources. Also, these two journals were classic journals in this field. The impact factors of the journals that published at least two of the 100 top-cited studies ranged from 1.344 to 70.670. We did not find a relationship between the number of studies published and journal impact factor. Also, the average number of citations for each article was not correlated with the journal impact factor. Similar results were reported in a previous study, which found that there were no statistically significant correlations between the number of top-cited studies and journal impact factors in vaccine (Zhang et al., 2019b). This phenomenon indicates that most researchers focus not only on the impact factor but also on the influence in their field when choosing journals in which to publish their research. The most frequently cited article received 24,252 citations and was published in *Neuropsychologia* in 1971 (Oldfield, 1971). This study proposed a simple and brief method for assessing handedness on a quantitative scale for use in neuropsychological, clinical, and experimental research. The results of this research were widely used in clinical research, and subsequent research used the results of this article for neuropsychological research. Coupled with the accumulation of time, this article had the highest frequency of citations.

The most productive country was the USA, with 60 publications. This was consistent with previous citation analyses for other disciplines (Shuaib et al., 2015; Zhang et al., 2019a). The most productive institute was the University of California, followed by the University of Pennsylvania, both from the USA. Thus, the USA made the greatest contribution to developments in neuropsychology in the specified time range. The main partners of the USA were England, Canada, and Netherlands. The pronounced influence of the USA may be attributed to its large number of scientific research institutions and abundant research funds. Indeed, we found that the top three most productive countries (the USA, England, and Canada) were all developed countries. Additionally, they had a cooperative relationship, which promoted the development of research in these countries. Although studies on neuropsychology continue to be conducted worldwide, developed countries may be more likely than developing countries to focus on this research topic. Therefore, there may be space for growth in terms of the quality and quantity of neuropsychology research in developing countries.

Of the 100 top-cited publications, 64 were original articles, and 36 were reviews. The average number of citations was higher for articles than for reviews. This finding indicates that neuropsychology researchers pay more attention to new findings in the field. The top three Web of Science categories were clinical neurology, behavioral sciences, and psychiatry. The clinical practice category had the highest number of neuropsychology articles. Neuropsychology is poised for a transformation in terms of concepts and methods due to advances in neuroimaging, the human genome project, psychometric theory, and information technology (Bilder, 2011). These findings may help researchers identify the most influential contributions to the field of neuropsychology, leading to ideas for future study.

There are some limitations to the present study. First, we only used the Web of Science database for our analysis and did not collect data from other databases, such as Scopus, Medline, and Google Scholar. Second, we only included research published in English. Third, over time, the number of citations of each publication will change, and so the 100 top-cited articles will also change. Fourth, we may have missed some studies that did not contain the term “neuropsycholog^∗^” in the title, abstract, author keywords, keywords, or journal name. Finally, citation rates are affected by many factors, many of which are beyond the scope of this study. In the future study, we will include more databases and dynamically track changes in these studies. Despite these limitations, we believe that as the first citation analysis in neuropsychology, our findings will contribute to the understanding of trends and classic publications in this field.

## Conclusion

In the present bibliometric study, we identified and analyzed the 100 top-cited publications in neuropsychology according to the authors, journal, publication year, country, institution, and study type. These data provide insight into the most impactful studies related to neuropsychology and will thus help doctors, researchers, and other stakeholders to enhance their understanding regarding the trends and influential contributions to neuropsychology research, fostering ideas for future study.

## Data Availability Statement

The original contributions presented in the study are included in the article/Supplementary Material, further inquiries can be directed to the corresponding author.

## Author Contributions

YoZ and YaZ designed the study. YaZ and YX analyzed the data. YaZ drafted the manuscript. YX, YC, LZ, and YoZ edited the manuscript. All authors approved the final version of the manuscript.

## Conflict of Interest

The authors declare that the research was conducted in the absence of any commercial or financial relationships that could be construed as a potential conflict of interest.

## Footnotes

1 http://www.vosviewer.com/
