# Supplementary material for: The 100 Top-Cited Studies on Neuropsychology: A Bibliometric Analysis
Source: Front Psychol. 2020 Nov 30;11:550716. doi: 10.3389/fpsyg.2020.550716 (PMC7734023; doi:10.3389/fpsyg.2020.550716)
Supplement: Supplementary file 1 [file Data_Sheet_1.docx]

**Supplementary materials**

Table S1. The 100 top-cited studies in neuropsychology

| Ranking | Title | Journal | Article type | Total citation | Citation per year* | Publication year | country |
| --- | --- | --- | --- | --- | --- | --- | --- |
| 1 | The assessment and analysis of handedness: the Edinburgh inventory | Neuropsychologia | Article | 24252 | 505 | 1971 | Scotland |
| 2 | Adolescence-limited and life-course-persistent antisocial-behavior - a developmental taxonomy | Psychological Review | Review | 5454 | 210 | 1993 | USA |
| 3 | The diagnosis of dementia due to Alzheimer's disease: Recommendations from the National Institute on Aging-Alzheimer's Association workgroups on diagnostic guidelines for Alzheimer's disease | Alzheimers & Dementia | Article | 5324 | 666 | 2011 | USA |
| 4 | Behavioral inhibition, sustained attention, and executive functions: Constructing a unifying theory of ADHD | Psychological Bulletin | Review | 3991 | 181 | 1997 | USA |
| 5 | The endophenotype concept in psychiatry: Etymology and strategic intentions | American Journal of Psychiatry | Review | 3669 | 229 | 2003 | USA |
| 6 | Vascular dementia - diagnostic-criteria for research studies - report of the ninds-airen international workshop | Neurology | Article | 3662 | 141 | 1993 | USA |
| 7 | Frontotemporal lobar degeneration - A consensus on clinical diagnostic criteria | Neurology | Article | 3617 | 172 | 1998 | England |
| 8 | Updating p300: An integrative theory of P3a and P3b | Clinical Neurophysiology | Review | 3443 | 287 | 2007 | USA |
| 9 | Separate visual pathways for perception and action | Trends in Neurosciences | Article | 3332 | 123 | 1992 | Canada |
| 10 | Insensitivity to future consequences following damage to human prefrontal cortex | Cognition | Article | 3288 | 132 | 1994 | USA |
| 11 | Toward defining the preclinical stages of Alzheimer's disease: Recommendations from the National Institute on Aging-Alzheimer's Association workgroups on diagnostic guidelines for Alzheimer's disease | Alzheimers & Dementia | Article | 3033 | 379 | 2011 | USA |
| 12 | Current concepts in mild cognitive impairment | Archives of Neurology | Article | 3031 | 168 | 2001 | USA |
| 13 | The episodic buffer: a new component of working memory? | Trends in Cognitive Sciences | Review | 2978 | 157 | 2000 | England |
| 14 | Physical basis of cognitive alterations in alzheimers disease - synapse loss is the major correlate of cognitive impairment | Annals of Neurology | Article | 2740 | 98 | 1991 | USA |
| 15 | The mini mental state examination - a comprehensive review | Journal of the American Geriatrics Society | Review | 2726 | 101 | 1992 | Canada |
| 16 | The consortium to establish a registry for alzheimers disease (CERAD) .1. clinical and neuropsychological assessment of alzheimers-disease | Neurology | Article | 2635 | 88 | 1989 | USA |
| 17 | Recent advances in the phencyclidine model of schizophrenia | American Journal of Psychiatry | Article | 2292 | 82 | 1991 | USA |
| 18 | The nature of recollection and familiarity: A review of 30 years of research | Journal of Memory and Language | Review | 2157 | 127 | 2002 | USA |
| 19 | The construction of autobiographical memories in the self-memory system | Psychological Review | Review | 2045 | 108 | 2000 | England |
| 20 | Episodic memory: From mind to brain | Annual Review of Psychology | Review | 1985 | 117 | 2002 | Canada |
| 21 | Sensitivity of revised diagnostic criteria for the behavioural variant of frontotemporal dementia | Brain | Article | 1884 | 236 | 2011 | USA |
| 22 | Frontal-subcortical circuits and human-behavior | Archives of Neurology | Review | 1621 | 62 | 1993 | USA |
| 23 | Vascular contributions to cognitive impairment and dementia a statement for healthcare professionals from the American Heart Association/American Stroke Association | Stroke | Review | 1609 | 201 | 2011 | USA |
| 24 | Stereotaxic display of brain lesions | Behavioural Neurology | Article | 1562 | 82 | 2000 | England |
| 25 | Cognitive dysfunction in multiple-sclerosis .1. frequency, patterns, and prediction | Neurology | Article | 1450 | 52 | 1991 | USA |
| 26 | Maternal thyroid deficiency during pregnancy and subsequent neuropsychological development of the child | New England Journal of Medicine | Article | 1424 | 71 | 1999 | USA |
| 27 | Silent brain infarcts and the risk of dementia and cognitive decline | New England Journal of Medicine | Article | 1411 | 88 | 2003 | Netherlands |
| 28 | Three parietal circuits for number processing | Cognitive Neuropsychology | Review | 1372 | 86 | 2003 | France |
| 29 | Practice parameter: Early detection of dementia: Mild cognitive impairment (an evidence-based review) - Report of the Quality Standards Subcommittee of the American Academy of Neurology | Neurology | Article | 1353 | 75 | 2001 | USA |
| 30 | The human hippocampus and spatial and episodic memory | Neuron | Review | 1281 | 75 | 2002 | England |
| 31 | Trail making test A and B: normative data stratified by age and education | Archives of Clinical Neuropsychology | Article | 1270 | 85 | 2004 | Canada |
| 32 | Hepatic encephalopathy-definition, nomenclature, diagnosis, and quantification: Final report of the working party at the 11th World Congresses of Gastroenterology, Vienna, 1998 | Hepatology | Article | 1268 | 75 | 2002 | Australia |
| 33 | Cognitive reserve | Neuropsychologia | Review | 1242 | 124 | 2009 | USA |
| 34 | Core systems of number | Trends in Cognitive Sciences | Review | 1239 | 83 | 2004 | USA |
| 35 | Long-term postoperative cognitive dysfunction in the elderly: ISPOCD1 study | Lancet | Article | 1231 | 59 | 1998 | Denmark |
| 36 | The functional neuroanatomy of the human orbitofrontal cortex: evidence from neuroimaging and neuropsychology | Progress in Neurobiology | Review | 1227 | 82 | 2004 | England |
| 37 | HIV-associated neurocognitive disorders persist in the era of potent antiretroviral therapy CHARTER Study | Neurology | Article | 1212 | 135 | 2010 | USA |
| 38 | Reorienting attention across the horizontal and vertical meridians - evidence in favor of a premotor theory of attention | Neuropsychologia | Article | 1201 | 38 | 1987 | Italy |
| 39 | The phonological loop as a language learning device | Psychological Review | Review | 1189 | 57 | 1998 | England |
| 40 | Differential behavioral effects in frontal lobe disease | Neuropsychologia | Article | 1186 | 23 | 1968 | USA |
| 41 | The American College of Rheumatology Nomenclature and Case Definitions for Neuropsychiatric Lupus Syndromes | Arthritis and Rheumatism | Review | 1178 | 59 | 1999 | USA |
| 42 | The role of prefrontal cortex in working-memory capacity, executive attention, and general fluid intelligence: An individual-differences perspective | Psychonomic Bulletin & Review | Review | 1177 | 69 | 2002 | USA |
| 43 | Cognitive deficit in 7-year-old children with prenatal exposure to methylmercury | Neurotoxicology and Teratology | Article | 1120 | 51 | 1997 | Denmark |
| 44 | Dorsal and ventral streams: a framework for understanding aspects of the functional anatomy of language | Cognition | Review | 1086 | 72 | 2004 | USA |
| 45 | A neuropsychological theory of positive affect and its influence on cognition | Psychological Review | Review | 1086 | 54 | 1999 | USA |
| 46 | Clinical and pathological diagnosis of Frontotemporal Dementia - Report of the work group on Frontotemporal Dementia and Pick's disease | Archives of Neurology | Article | 1063 | 59 | 2001 | USA |
| 47 | Functional specialization for semantic and phonological processing in the left inferior prefrontal cortex | Neuroimage | Article | 1060 | 53 | 1999 | USA |
| 48 | Preclinical evidence of Alzheimer's disease in persons homozygous for the epsilon 4 allele for apolipoprotein E | New England Journal of Medicine | Article | 1018 | 44 | 1996 | USA |
| 49 | Toward a theory of episodic memory: The frontal lobes and autonoetic consciousness | Psychological Bulletin | Review | 1010 | 46 | 1997 | Canada |
| 50 | 'Oops!': Performance correlates of everyday attentional failures in traumatic brain injured and normal subjects | Neuropsychologia | Article | 996 | 45 | 1997 | England |
| 51 | Prediction of AD with MRI-based hippocampal volume in mild cognitive impairment | Neurology | Article | 992 | 50 | 1999 | USA |
| 52 | Remembering the past and imagining the future: Common and distinct neural substrates during event construction and elaboration | Neuropsychologia | Article | 985 | 82 | 2007 | USA |
| 53 | Depression duration but not age predicts hippocampal volume loss in medically healthy women with recurrent major depression | Joural of Neuroscience | Article | 984 | 49 | 1999 | USA |
| 54 | Effect on parkinsonian signs and symptoms of bilateral subthalamic nucleus stimulation | Lancet | Article | 981 | 41 | 1995 | France |
| 55 | Cognitive impairment in multiple sclerosis | Lancet Neurology | Review | 979 | 89 | 2008 | USA |
| 56 | Neuropsychological function in schizophrenia - selective impairment in memory and learning | Archives of General Psychiatry | Article | 968 | 35 | 1991 | USA |
| 57 | Diagnostic criteria for mild cognitive impairment in Parkinson's disease: Movement Disorder Society Task Force guidelines | Movement Disorders | Article | 947 | 135 | 2012 | USA |
| 58 | Cumulative effects associated with recurrent concussion in collegiate football players - The NCAA Concussion Study | JAMA-Journal of the American Medical Association | Article | 944 | 59 | 2003 | USA |
| 59 | Deficits on subject-ordered tasks after frontal-lobe and temporal-lobe lesions in man | Neuropsychologia | Article | 936 | 25 | 1982 | Canada |
| 60 | Executive function and the frontal lobes: A meta-analytic review | Neuropsychology Review | Review | 934 | 72 | 2006 | USA |
| 61 | Rapid automatized naming (RAN) - dyslexia differentiated from other learning-disabilities | Neuropsychologia | Article | 933 | 22 | 1976 | USA |
| 62 | Grasping objects - the cortical mechanisms of visuomotor transformation | Trends in Neurosciences | Review | 931 | 39 | 1995 | France |
| 63 | Reading the mind in cartoons and stories: an fMRI study of 'theory of mind' in verbal and nonverbal tasks | Neuropsychologia | Article | 921 | 48 | 2000 | England |
| 64 | Planning and spatial working memory following frontal-lobe lesions in man | Neuropsychologia | Article | 910 | 31 | 1990 | England |
| 65 | Word fluency and brain damage | Neuropsychologia | Article | 910 | 18 | 1967 | USA |
| 66 | The repeatable battery for the assessment of neuropsychological status (RBANS): Preliminary clinical validity | Journal of Clinical and Experimental Neuropsychology | Article | 902 | 43 | 1998 | USA |
| 67 | Atrophy of medial temporal lobes on MRI in probable alzheimers-disease and normal aging - diagnostic-value and neuropsychological correlates | Journal of Neurology Neurosurgery and Psychiatry | Article | 895 | 33 | 1992 | Netherlands |
| 68 | Acute effects and recovery time following concussion in collegiate football players - The NCAA Concussion Study | JAMA-Journal of the American Medical Association | Article | 891 | 56 | 2003 | USA |
| 69 | National Institute of Neurological Disorders and Stroke-Canadian Stroke Network vascular cognitive impairment harmonization standards | Stroke | Review | 873 | 67 | 2006 | USA |
| 70 | Neuropsychological deficits in neuroleptic naive patients with first-episode schizophrenia | Archives of General Psychiatry | Article | 861 | 34 | 1994 | USA |
| 71 | Long-term cognitive impairment after Critical Illness | New England Journal of Medicine | Article | 854 | 142 | 2013 | USA |
| 72 | Prevalence and characteristics of dementia in Parkinson disease - An 8-year prospective study | Archives of Neurology | Article | 849 | 53 | 2003 | Norway |
| 73 | Amyloid beta deposition, neurodegeneration, and cognitive decline in sporadic Alzheimer's disease: a prospective cohort study | Lancet Neurology | Article | 844 | 141 | 2013 | Australia |
| 74 | What is "special" about face perception? | Psychological Review | Article | 843 | 40 | 1998 | USA |
| 75 | Improving lesion-symptom mapping | Journal of Cognitive Neuroscience | Article | 837 | 70 | 2007 | USA |
| 76 | Assessment and development of executive function (EF) during childhood | Child Neuropsychology | Article | 836 | 49 | 2002 | Australia |
| 77 | Hippocampus: Cognitive processes and neural representations that underlie declarative memory | Neuron | Review | 822 | 55 | 2004 | USA |
| 78 | Depression and parkinsons-disease - a review | American Journal of Psychiatry | Article | 822 | 30 | 1992 | USA |
| 79 | Dissociable neural responses to facial expressions of sadness and anger | Brain | Article | 817 | 41 | 1999 | England |
| 80 | The effects of clozapine, risperidone, and olanzapine on cognitive function in schizophrenia | Schizophrenia Bulletin | Review | 812 | 41 | 1999 | USA |
| 81 | A normative developmental-study of executive function - A window on prefrontal function in children | Developmental Neuropsychology | Article | 812 | 29 | 1991 | USA |
| 82 | Cytokine-associated emotional and cognitive disturbances in humans | Archives of General Psychiatry | Article | 806 | 45 | 2001 | Israel |
| 83 | The fusiform face area: a cortical region specialized for the perception of faces | Philosophical Transactions of the Royal Society B-Biological Sciences | Review | 803 | 62 | 2006 | USA |
| 84 | Further analysis of hippocampal amnesic syndrome - 14-year follow-up study of HM | Neuropsychologia | Article | 793 | 16 | 1968 | Canada |
| 85 | HIV-associated neurocognitive disorders before and during the era of combination antiretroviral therapy: differences in rates, nature, and predictors | Journal of Neurovirology | Article | 792 | 99 | 2011 | USA |
| 86 | Executive functioning as a predictor of children's mathematics ability: Inhibition, switching, and working memory | Developmental Neuropsychology | Article | 787 | 44 | 2001 | Scotland |
| 87 | The mental deterioration battery: Normative data, diagnostic reliability and qualitative analyses of cognitive impairment | European Neurology | Article | 785 | 34 | 1996 | Italy |
| 88 | Quantitative brain magnetic resonance imaging in attention-deficit hyperactivity disorder | Archives of General Psychiatry | Review | 784 | 34 | 1996 | USA |
| 89 | Hopkins Verbal Learning Test Revised: Normative data and analysis of inter-form and test-retest reliability | Clinical Neuropsychologist | Article | 779 | 37 | 1998 | USA |
| 90 | The role of the posterior cingulate cortex in cognition and disease | Brain | Review | 770 | 154 | 2014 | England |
| 91 | Identification of separable cognitive factors in schizophrenia | Schizophrenia Research | Article | 766 | 51 | 2004 | USA |
| 92 | Imaging beta-amyloid burden in aging and dementia | Neurology | Article | 764 | 64 | 2007 | Australia |
| 93 | Left ventrolateral prefrontal cortex and the cognitive control of memory | Neuropsychologia | Review | 763 | 64 | 2007 | USA |
| 94 | Pseudoneglect: A review and meta-analysis of performance factors in line bisection tasks | Neuropsychologia | Review | 763 | 40 | 2000 | USA |
| 95 | Cognitive function across manic or hypomanic, depressed, and euthymic states in bipolar disorder | American Journal of Psychiatry | Article | 758 | 51 | 2004 | Spain |
| 96 | Cognitive deficits in depression - Possible implications for functional neuropathology | British Journal of Psychiatry | Review | 755 | 42 | 2001 | Australia |
| 97 | The functional neuroanatomy of autobiographical memory: A meta-analysis | Neuropsychologia | Review | 739 | 57 | 2006 | Canada |
| 98 | The elusive nature of executive functions: A review of our current understanding | Neuropsychology Review | Review | 738 | 62 | 2007 | USA |
| 99 | Two visual systems re-viewed | Neuropsychologia | Article | 737 | 67 | 2008 | England |
| 100 | Morphometric study of human cerebral-cortex development | Neuropsychologia | Article | 736 | 25 | 1990 | USA |

*: The annual average number of citations of the 100 articles was correlated with the total number of citations (*r* = 0.676, *P < 0.01*).


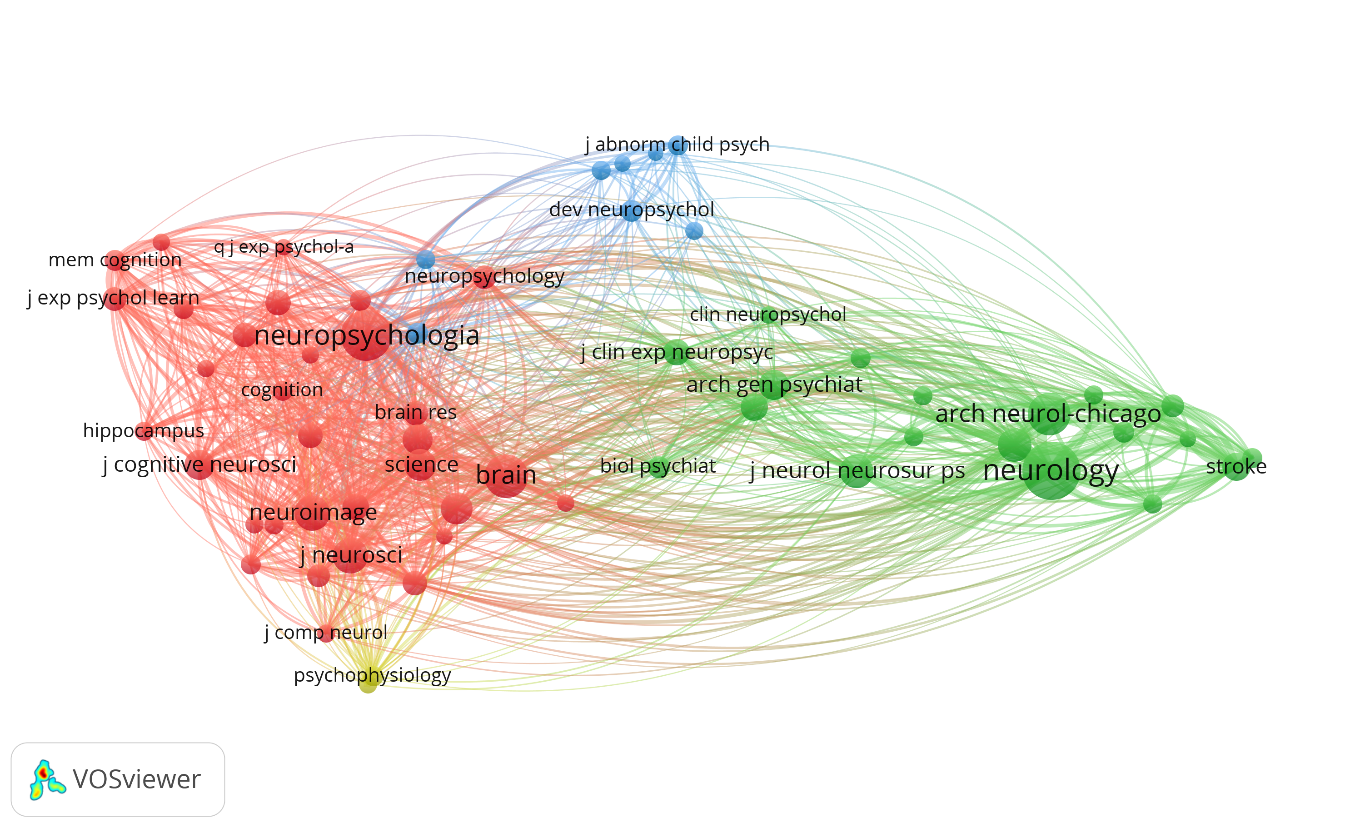


Figure S1: The co-citation map of cited sources. Different colors indicate different research areas and the size of circles represents the counts of co-citations. The distance between the two circles indicates their correlation.


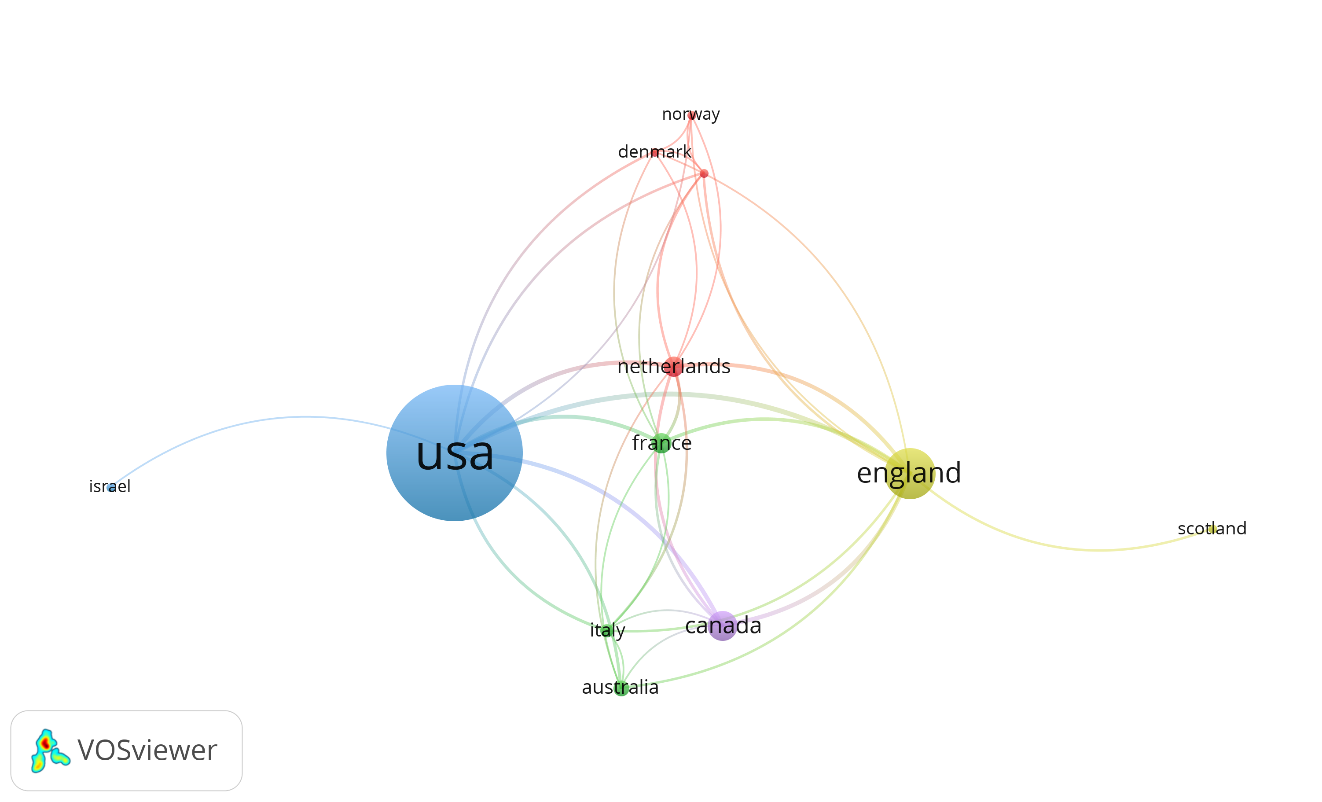


Figure S2: The co-authorship map of countries. Different colors indicate different clusters and the size of circles indicates the number of publications. The thickness of the lines represents the link strength of the countries.


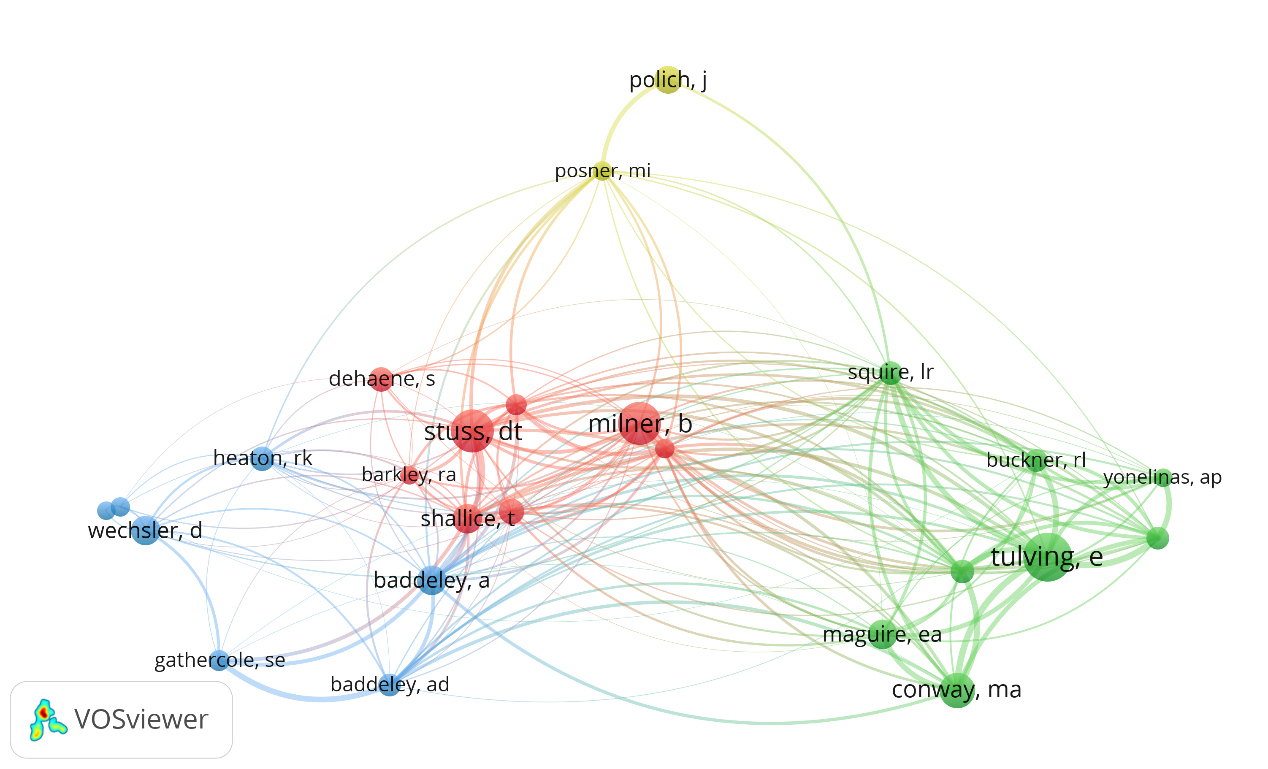


Figure S3: The co-citation map of authors. Different colors indicate different research areas and the size of circles represents the counts of co-citations. The distance between the two circles indicates their correlation.
